# Expression of Serum Exosomal and Esophageal MicroRNA in Rat Reflux Esophagitis

**DOI:** 10.3390/ijms18081611

**Published:** 2017-07-25

**Authors:** Risa Uemura, Yoshiki Murakami, Atsushi Hashimoto, Akinari Sawada, Koji Otani, Koichi Taira, Shuhei Hosomi, Yasuaki Nagami, Fumio Tanaka, Noriko Kamata, Hirokazu Yamagami, Tetsuya Tanigawa, Toshio Watanabe, Y-h Taguchi, Yasuhiro Fujiwara

**Affiliations:** 1 Department of Gastroenterology, Osaka City University Graduate School of Medicine, Osaka 545-8585, Japan; uemurarisa@med.osaka-cu.ac.jp (R.U.); m2075060@med.osaka-cu.ac.jp (A.H.); m1164972@med.osaka-cu.ac.jp (A.S.); kojiotani@med.osaka-cu.ac.jp (K.O.); koichit0802@gmail.com (K.T.); m1265271@med.osaka-cu.ac.jp (S.H.); yasuaki1975@gmail.com (Y.N.); m2079981@med.osaka-cu.ac.jp (F.T.); nkamata@med.osaka-cu.ac.jp (N.K.); yamagami@med.osaka-cu.ac.jp (H.Y.); ttanigawa@med.osaka-cu.ac.jp (T.T.); watanabet@med.osaka-cu.ac.jp (T.W.); 2 Department of Hepatology, Osaka City University Graduate School of Medicine, Osaka 545-8585, Japan; yoshimurak@med.osaka-cu.ac.jp; 3 Department of Physics, Chuo University, Tokyo 112-8551, Japan; tag@granular.com

**Keywords:** gastroesophageal reflux disease, non-erosive reflux disease, reflux esophagitis, microRNA, exosome

## Abstract

Gastroesophageal reflux disease (GERD) is a common upper gastrointestinal disease. However, the role of exosomal microRNAs (miRNAs) and esophageal miRNAs in GERD has not been studied. A rat model of acid reflux esophagitis was used to establish a novel diagnosis marker for GERD and examine dynamics of miRNA expression in GERD. Rats were sacrificed 3 (acute phase), 7 (sub-acute phase) and 21 days (chronic phase) after induction of esophagitis. Exosomes were extracted from serum, and the expression patterns of serum miRNAs were analyzed. Four upregulated miRNAs (miR-29a-3p, 128-3p, 223-3p and 3473) were identified by microarray analysis. The expression levels of exosomal miR-29a-3p were significantly higher in the chronic phase of reflux esophagitis compared with controls, and increased expression of miR-29a-3p was specific to chronic reflux esophagitis. Esophageal miR-223-3p expression was higher compared with controls, and gradually decreased from acute to chronic phase in esophagitis. In conclusion, exosomal miR-29a-3p and esophageal miR-223-3p might play roles in GERD.

## 1. Introduction

Gastroesophageal reflux disease (GERD) is a common upper gastrointestinal disease that is divided into two subtypes: erosive reflux disease (ERD) and non-erosive reflux disease (NERD), the latter being defined as the presence of troublesome reflux symptoms without esophageal mucosal breakage [1]. Although the first-line treatment for GERD is a proton pump inhibitor (PPI) [2], approximately 20–40% of GERD patients are refractory to PPI therapy, especially NERD patients [3]. One reason for the high incidence of PPI-resistance in NERD is its heterogeneous pathogenesis, including abnormal esophageal acid exposure and esophageal hypersensitivity. Recently, the Rome IV criteria proposed the concept of reflux hypersensitivity and functional heartburn in esophageal functional disorders, and these two diseases might include NERD in clinical settings [4]. However, it is difficult to differentiate between GERD and reflux hypersensitivity or functional heartburn without using esophageal impedance-pH monitoring [5]. Simple biomarkers of GERD are necessary to replace invasive diagnostic modalities.

MicroRNAs (miRNAs) are small, non-coding RNAs that control gene expression by annealing to complementary target mRNAs [6,7]. Specific miRNAs participate in multiple cellular functions and events, including cell generation, apoptosis, differentiation, multiplication, metabolism, and carcinogenesis [8]. The identification of circulating miRNAs in the serum exosomes, which are membrane-bound vesicles that are 40–100 nm in diameter and secreted by various cells, indicated that miRNAs may be potentially useful as clinical diagnostic or prognostic tools [9,10,11,12].

There have been several studies about exosomal miRNAs in esophageal cancer [13,14]. However, the role of exosomal miRNAs and esophageal miRNAs in GERD has not been reported yet. The aim of this study was to observe changes of exosomal and esophageal miRNAs to establish novel diagnosis markers for GERD and to examine dynamics of miRNA expression using a rat model.

## 2. Results

### 2.1. Macroscopic and Histological Findings

**Figure 1:**
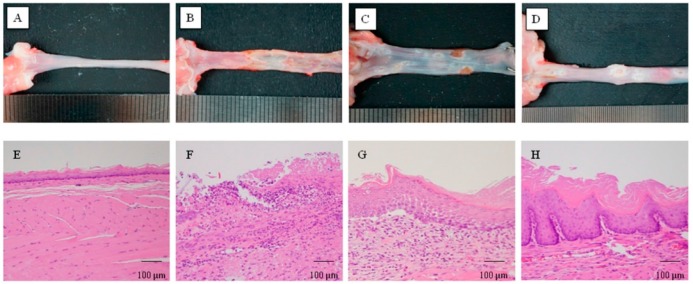
Macroscopic appearance and histological findings of rat esophagitis. (**A**,**E**) Normal esophagus examination revealed thin squamous cell epithelium and few inflammatory cells; (**B**,**F**) Erosions and ulcers with marked inflammatory cell infiltration were observed on day 3 after inducing reflux esophagitis; (**C**,**G**) Size of ulcers and erosions, and number of inflammatory cells were decreased on day 7 after inducing reflux esophagitis; (**D**,**H**) Whitish lesions at the middle and lower part of the esophagus, and histological mucosal thickening with elongation of the papilla, basal cell hyperplasia and inflammatory cell infiltration were found on day 21 after induction of reflux esophagitis. (**A**–**D**) Macroscopic analysis; (**E**–**H**) Hematoxylin and eosin staining.

Figure 1A shows the macroscopic appearance of the normal esophagus. The normal esophagus had a thin esophageal epithelium with few inflammatory cells in the submucosa (Figure 1E). In contrast, there were several large ulcers with edematous mucosa on day 3 after inducing esophagitis (Figure 1B). Histological examination revealed defects in the epithelial layer and marked infiltration of inflammatory cells in the lamina propria, submucosa, and the bases of the ulcers (Figure 1F). Esophageal ulcers with edematous mucosa and inflammatory cell infiltration were decreased on day 7 compared with those observed on day 3 (Figure 1C,G). Several whitish esophageal lesions associated with marked thickening of the esophageal wall were found in the middle and lower part of the esophagitis on day 21 (Figure 1D). Histological examination revealed mucosal thickening with basal cell hyperplasia and infiltration of inflammatory cells to the lamina propria and submucosa (Figure 1H). The histological findings on day 21 were consistent with those observed in the patients with reflux esophagitis. We confirmed these macroscopic and histological changes in all rats with reflux esophagitis.

### 2.2. Unique Expression Pattern of Exosomal miRNA in Reflux Esophagitis

**Figure 2:**
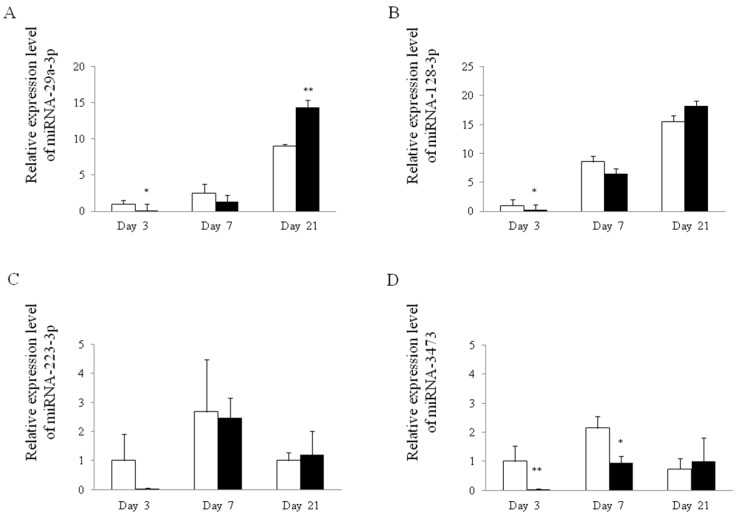
Expression of exosomal microRNA (miRNA) in rat reflux esophagitis. Relative expression of miRNA-29a-3p (**A**); miRNA-128-3p (**B**); miRNA-223-3p (**C**); and miRNA-3473 (**D**) in serum exosomes from rats with esophagitis compared to control rats. White bars represent control and closed bars represent reflux esophagitis. * *p* < 0.01 versus control, ** *p* < 0.05 versus control.

Exosomes were extracted from serum and the expression patterns of serum miRNAs on day 21 were analyzed via microarray. Microarray analysis revealed an upregulation of miR-29a-3p, miR-128-3p, miR-223-3p and miR-3473 in reflux esophagitis (*p* < 0.05 compared to controls). Then, we verified the expression pattern of exosomal miRNAs by real-time RT-PCR. The expression levels of these miRNA are presented as ratios to the mean value in exosomal miRNA of the control on day 3. The expression level of exosomal miR-29a-3p in reflux esophagitis was significantly lower on day 3, but significantly higher (2.4-fold) on day 21 compared with controls (Figure 2A). Analogously, the expression level of exosomal miR-128-3p in reflux esophagitis was significantly lower on day 3 compared with the control, but there was no significant difference on day 7 and 21 between control and reflux esophagitis (Figure 2B). There were no differences in miR-223-3p between control and reflux esophagitis during any phase (Figure 2C). The expression level of exosomal miR-3473 in reflux esophagitis was significantly lower on day 3 and 7 but not on day 21 compared with the control (Figure 2D). These results revealed that only miR-29a-3p expression significantly increased in the chronic phase of reflux esophagitis, suggesting exosomal miR-29a-3p might be a novel candidate surrogate marker for chronic reflux esophagitis.

### 2.3. The Specificity of Exosomal miR-29a-3p with Reflux Esophagitis

**Figure 3:**
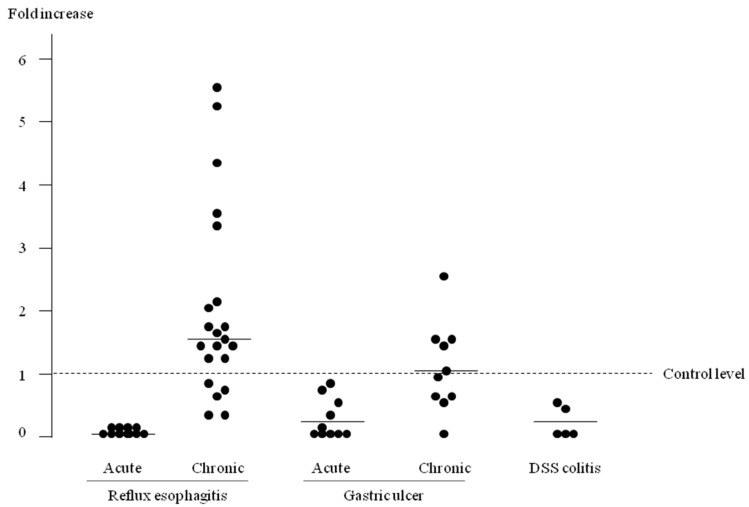
Relative expression level of exosomal microRNA (miRNA)-29a-3p in different rat mucosal injury models. Increases in exosomal miRNA-29a-3p expression were specific to chronic reflux esophagitis. Bars represent mean level of exosomal miRNA-29a-3p in each model. DSS, Dextran sulfate sodium-induced colitis.

Next, we examined exosomal miRNA expression in other animal models of gastrointestinal disease to determine whether increased expression of exosomal miR-29a-3p was specific to chronic reflux esophagitis. The relative expression level of exosomal miR-29a-3p was presented as ratios to the mean value of the control in each model (Figure 3). Increased expression level of exosomal miR-29a-3p was specific to chronic reflux esophagitis compared with gastric ulcers and dextran sulfate sodium (DSS)-colitis models. The expression of exosomal miR-29a-3p in the acute phase of reflux esophagitis, gastric ulcer and colitis was significantly lower compared with the control.

### 2.4. miRNA and mRNA Expression Analysis in Esophageal Tissue by Real-Time RT-PCR

**Figure 4:**
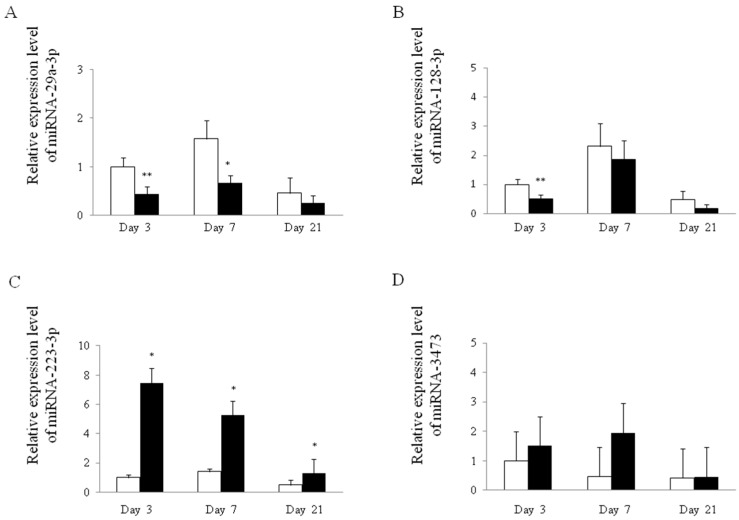
Expression of esophageal microRNA (miRNA) in rat reflux esophagitis. Relative expression of miRNA-29a-3p (**A**); miRNA-128-3p (**B**); miRNA-223-3p (**C**) and miRNA-3473 (**D**) in esophageal tissue from rats with esophagitis compared to control rats. White bars represent control and closed bars represent reflux esophagitis. * *p* < 0.01 versus control, ** *p* < 0.05 versus control.

**Figure 5:**
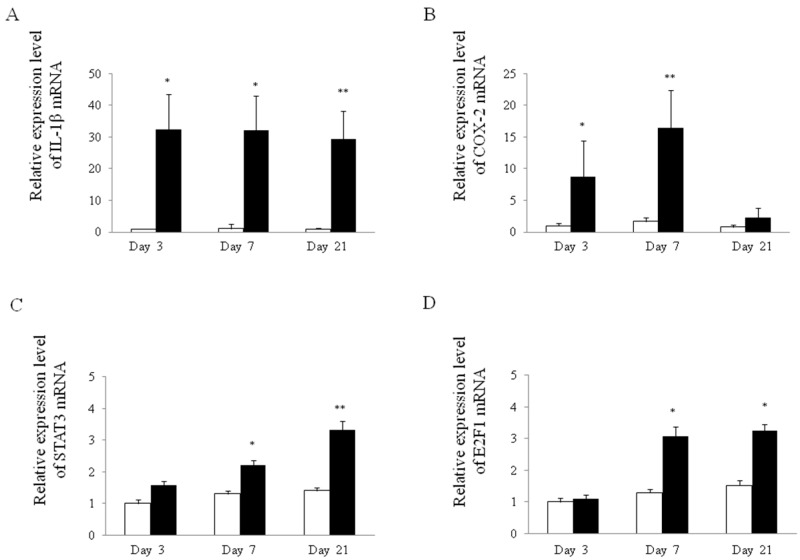
Expression of esophageal mRNA in rat esophagitis. Relative mRNA expression of *IL-1β* (**A**); *COX-2* (**B**); *STAT3* (**C**) and *E2F1* (**D**) in esophagus tissue of rats with or without esophagitis. White bars represent control and closed bars represent reflux esophagitis. * *p* < 0.01 versus control, ** *p* < 0.05 versus control.

Figure 4 and Figure 5 show the expression levels of miRNA and mRNA in esophageal tissue. The expression levels of these miRNA and mRNA are presented as ratios to the mean value in control tissue on day 3. The expression levels of miR-29a-3p and miR-128-3p in reflux esophagitis were significantly lower than those of the controls on day 3 (Figure 4A,B). The expression patterns of miR-29a-3p, miR-128-3p and miR-3473 were not dramatically changed during acute to chronic phases (Figure 4A,B,D). The expression level of miR-223-3p on day 3 in reflux esophagitis was significantly higher compared with controls and gradually decreased from the acute phase to the chronic phase (Figure 4C).

The expression level of *interleukin-1β* (*IL-1β*) in reflux esophagitis was significantly higher than the control in all phases examined (Figure 5A). The expression level of *cyclooxygenase-2* (*COX-2*) in reflux esophagitis was significantly higher than the level in control tissue on day 3 and 7, but not day 21 (Figure 5B). On the other hand, the expression levels of *transcription 3* (*STAT3*) and *E2F1* in reflux esophagitis were significantly higher than the control on both day 7 and 21. The expression patterns of *STAT3* and *E2F1* were gradually increased in a time-dependent manner (Figure 5C,D).

### 2.5. The Relationship between miR-223-3p and mRNA in Esophageal Tissue

**Figure 6:**
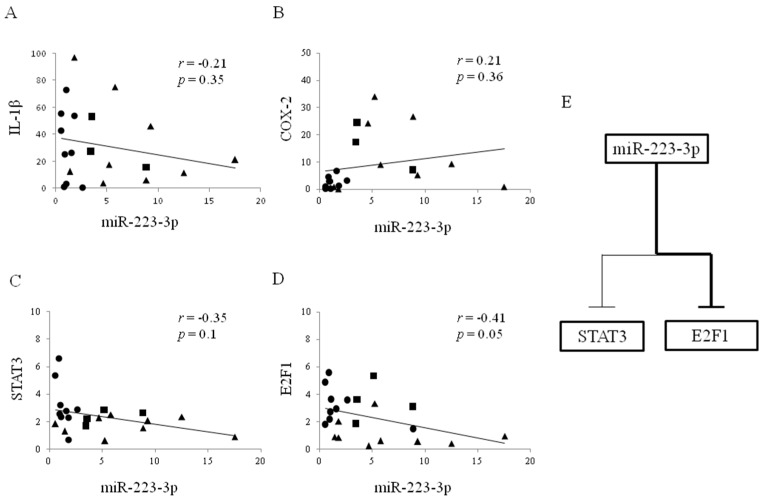
Correlation between microRNA-223-3p and mRNA expression in rat reflux esophagitis. (**A**–**D**) Correlations between expression of miR-223-3p and *IL-1β*, *COX-2*, *STAT3*, or *E2F1*. Reflux esophagitis on day 3 ▲, day 7 ■ and day 21 ●; (**E**) Scheme of the association between miR-223-3p and *E2F1* or *STAT3*; miR-223-3p mainly regurated *E2F1* (the bold line) compared to *STAT3* (the thin line).

Figure 6A–D shows the correlation between expression level of miR-223-3p and other mRNAs in esophageal tissue. There was a trend for negative correlation between miR-223-3p and *E2F1* (*r* = −0.41, *p* = 0.05) and a weak negative correlation between miR-223-3p and *STAT3* (*r* = −0.35). There were no correlations between miR-223-3p and *IL-1β* or *COX-2*. Therefore, the data suggest that miR-223-3p may inversely regulate *E2F1* or *STAT3* (Figure 6E).

## 3. Discussion

Several studies have reported the role of miRNA in esophageal diseases, especially esophageal squamous cell carcinoma (ESCC). Tanaka et al. demonstrated that serum exosomal miR-21 was upregulated in patients with ESCC compared with those who have benign diseases without systemic inflammation. They found that the expression level of exosomal miR-21 was positively correlated with tumor progression and prognosis [14]. In another previous report, plasma miR-18a was found as a possible useful biomarker for diagnosis and monitoring in patients with ESCC [13]. Although many studies have shown the potential role of miRNAs as biomarkers for diagnosis and prognosis of ESCC, there are few reports about the role of miRNA in GERD. Yan et al. reported that miR-203 expression in tongue exfoliated cells was significantly downregulated in GERD patients compared to controls [15], suggesting that miR-203 testing in tongue coating samples might assist with GERD diagnosis. However, no study has yet evaluated serum exosomal and esophageal miRNAs in GERD.

This is the first study about the association between GERD and exosomal or esophageal miRNA. Our study has two strengths. First, we found that only the exosomal miR-29a-3p level was significantly increased in chronic reflux esophagitis among four miRNAs identified by microarray analysis. Increases in serum exosomal miR-29a-3p were specific to chronic reflux esophagitis since exosomal miR-29a-3p was not increased in other gastrointestinal mucosal injury models, including gastric ulcers and colitis. These results suggest that serum exosomal miR-29a-3p might be a specific marker for GERD. The expression of exosomal miR-29a-3p in the acute phase of reflux esophagitis, gastric ulcer and colitis was significantly lower compared with the control. However, the reason why exosomal miR-29a-3p was downregulated in the acute phase was not clear.

Precise mechanisms of increased expression of exosomal miR-29a-3p in chronic reflux esophagitis, as well as the discrepancy of miR-29a-3p expression between serum exosomes and esophageal tissue, are unknown. There is the possibility of a time difference between expression in serum exosomes and esophageal tissue. The miR-29 group is composed of miR-29a, 29b-1, 29b-2, and 29c. MicroRNA-29a and 29b-1 are processed from chromosome 7, and miR-29b-2 and 29c are transcribed from chromosome 1. Since forced overexpression of miR-29 markedly suppresses collagen 1 A1 mRNA and protein expression [16,17], miR-29 plays an anti-fibrotic role in the liver and other organs [18]. However, our results showed that fibrosis was not observed and esophageal miR-29a-3p did not increase in the chronic phase of reflux esophagitis.

Second, we identified that the expression of esophageal miR-223-3p in reflux esophagitis was significantly increased compared with control tissue, and expression gradually decreased during the acute to chronic phases of reflux esophagitis. Several studies reported that miR-223 impacts several different cellular processes, including cell cycle regulation, invasiveness, hematopoietic differentiation and immune function. MicroRNA-223 is deregulated in many inflammation-related disorders including a mouse inflammatory bowel disease model [19,20]. Our study showed that the expression of miR-223-3p was inversely related to the expression of *E2F1* or *STAT3* in esophageal tissue. This result is further supported by other studies [21,22,23,24].

The roles of *E2F1* and *STAT3* in chronic reflux esophagitis should be discussed. Although *E2F1* plays a role in promoting esophageal carcinogenesis, it regulates several genes associated with the cell cycle [21,22]. Therefore, increased expression of *E2F1* might be related to basal cell hyperplasia in chronic reflux esophagitis. *STAT3* regulates *IL-6* genes and expression of *STAT3* increases during the transition from Barrett’s metaplasia to adenocarcinoma [23,24]. We did not examine Barrett’s metaplasia in this study, but our previous study showed that long-term reflux caused Barrett’s esophagus with increased expression of *Th-2* cytokines [25]. *STAT3* might be associated with early events of Barrett’s esophagus. Further studies are required.

## 4. Materials and Methods

### 4.1. Animals and Induction of Esophagitis, Gastric Ulcers and Colitis

Specific pathogen-free male Wistar rats (Japan SLC, Hamamatsu, Japan) weighing approximately 200 g at the start of the experiment were used. They were housed at a constant temperature (22 ± 2 °C) in cages with an automatically controlled 12 h light/dark cycle (light on at 9:00 a.m.) and they had free access to food and water. Acid reflux esophagitis was induced by the methods described by Omura et al. [26]. In brief, the duodenum near the pyloric ring was covered with a 2 mm-wide piece of 18 Fr Nelaton catheter (Terumo Co, Tokyo, Japan), and the transitional region between the forestomach and the glandular portion was ligated to enhance reflux of gastric contents into the esophagus. Solid food was withdrawn for two days after induction of esophagitis but rats were allowed drinking water. Rats were sacrificed 3 (acute phase), 7 (sub-acute phase) and 21 days (chronic phase) after inducing esophagitis. In the gastric ulcer model of rats, acetic acid (80 µL) was applied to the serosa of the glandular stomach at the anterior wall through a polyethylene tube (6.0 mm inner diameter) for 90 s as described previously [27,28]. Rats were sacrificed 2 (acute phase) and 14 days (chronic phase) after injecting acetic acid. Colitis was produced in rats by providing drinking water containing synthetic 5% dextran sulfate sodium (DSS, Wako Chemical, Miyazaki, Japan) [29] and rats were sacrificed on day 7. Sham-operated rats were used as controls for reflux esophagitis and gastric ulcers. On the other hand, control animals for DSS-colitis received the vehicle alone. Blood samples from rats were collected by puncture during terminal anesthesia and centrifuged at 1500× *g* for 10 min at 4 °C. The supernatants were transferred to fresh tubes and stored at −80 °C until analysis. Esophageal tissues were excised and immediately stored in RNAlater solution (Applied Biosystems, Foster, CA, USA). For histological analysis, samples were gently rinsed with saline and fixed in 10% buffered formalin. Samples were embedded in paraffin and 4-μm thick sections were prepared. Hematoxylin and eosin staining was performed for standard morphological analysis. All experimental procedures were approved by the Animal Care Committee of the Osaka City University Graduate School of Medicine (Approval number 14008, 3 September, 2014).

### 4.2. Isolation of Serum Exosomes and RNA Extraction

Serum exosomes were isolated with Exoquick according to the manufacturer’s protocol (System Biosciences, Mountain View, CA, USA) [30]. Briefly, blood was obtained by cardiac puncture and 900 µL of serum was collected and mixed with Exoquick. Samples were centrifuged at 1500× *g* for 30 min followed by incubation overnight at 4 °C. The supernatant was decanted and the exosome pellet was resuspended in phosphate-buffered saline. Total RNA was extracted using the miRNeasy kit (Qiagen, Hilden, Germany).

### 4.3. Microarray Analysis

To detect exosomal miRNA, 100 ng of RNA was labeled and hybridized using the Rat miRNA Microarray, 8 × 15 K Rel.19.0 (Agilent Technologies, Santa Clara, CA, USA). Hybridization signals were detected with the Agilent DNA microarray scanner G2539A and the scanned images were analyzed using Agilent feature extraction software (v10.10.1.1). Data were analyzed using Scan Control (ver A.8.5.1) software (Agilent Technologies) and normalized as follows: (i) Values below 0.01 were set to 0.01; (ii) Each measurement was divided by the 75th percentile of all measurements from the same species. All data were deposited in NCBI’s Gene Expression Omnibus and are accessible through GEO Series accession number GSE94719.

### 4.4. Real-Time qPCR for miRNA

TaqMan microRNA assays (Applied Biosystems) were used to quantify the relative expression level of hsa-miR-29a-3p (assay ID 002112), hsa-miR-128-3p (assay ID 002216), hsa-miR-223-3p (assay ID 000526) and rno-miR-3473 (assay ID. 475642). U6 snRNA (assay ID 001973) was used as an internal control. cDNA was synthesized using the Taqman miRNA RT Kit (Applied Biosystems). Total RNA (10 ng/mL) in 5 µL of nuclease-free water was added to 3 µL of 5× RT primer, 1.5 µL of 10× reverse transcriptase buffer, 0.15 µL of 100 mM dNTP, 0.19 µL of RNase inhibitor, 4.16 µL of nuclease-free water and 50 U of reverse transcriptase in a total volume of 15 µL. The reaction was performed in triplicate for 30 min at 16 °C, 30 min at 42 °C and 5 min at 85 °C. Analyses were performed using an Applied Biosystems 7500 Fast Real-Time PCR system and software (Thermo Fisher Scientific Inc., Waltham, MA, USA). The reaction mixture was prepared using the TaqMan Fast Universal PCR master mixture (Thermo Fisher Scientific Inc.). Thermal cycling conditions were as follows: 45 cycles of 95 °C for 15 s and 60 °C for 1 min.

### 4.5. Real-Time qPCR for mRNA

Total RNA was isolated from esophageal tissue using an ISOGEN kit (Nippon Gene Co., Ltd., Tokyo, Japan). Complementary DNA was produced using the High Capacity RNA-to-cDNA Kit (Thermo Fisher Scientific Inc.). Real-time quantitative RT-PCR analyses were performed using an Applied Biosystems 7500 Fast Real-Time PCR system and software (Thermo Fisher Scientific Inc.). The reaction mixture was prepared according to the manufacturer’s protocol using the TaqMan Fast Universal PCR master mixture (Thermo Fisher Scientific Inc.). Thermal cycling conditions were as follows: 45 cycles of 95 °C for 15 s and 60 °C for 1 min. The expression levels of *IL-1β*, *COX-2*, signal transducer and activator of *STAT3* and *E2F1* were quantified in esophageal tissue using real-time RT-PCR and standardized to TaqMan *glyceraldehyde-3-phosphate dehydrogenase* (*GAPDH*; Thermo Fisher Scientific Inc.) mRNA levels. The primer sequences were as follows: *IL-1β* Forward: 5′-CACCTCTCAAGCAGAGCACAG-3′, Reverse: 5′-GGGTTCCATGGTGAAGTCAAC-3′, *COX-2* Forward: 5′-CATGATCTACCCTCCCACG-3′, Reverse: 5′-CAGACCAAAGACTTCCTGCCC-3′, *STAT3* Forward: 5′-ACCCGCCAACAAATTAAGAAACTG-3′, Reverse: 5’-CACCACGAAGGCACTCTTCATTA-3′, and *E2F1* Forward: 5′-CATATCCAGTGGCTAGGCAGC-3′, Reverse: 5′-GCTCACTCTCCTGCAGTTGTT-3′.

### 4.6. Statistical Analyses

Statistical analyses were performed using a Student’s *t*-test. *p* values less than 0.05 were considered statistically significant. Data were expressed as mean ± standard error (SE). Associations between expression levels of miRNA and mRNA were analyzed by Pearson’s correlation statistics. Microarray data were statistically analyzed using ANOVA or Welch’s test and Bonferroni correction for multiple hypotheses testing. The statistical analysis was performed with SPSS (22.0J SPSS Japan, Tokyo, Japan).

## 5. Conclusions

In conclusion, our results demonstrated that exosomal miR-29a-3p might assist in the diagnosis of GERD, and increased expression of esophageal miR-223-3p was inversely associated with expression of *E2F1* or *STAT3* in esophageal tissue reflux esophagitis.

## Abbreviations

GERD	Gastroesophageal reflux disease
ERD	Erosive reflux disease
NERD	Non-erosive reflux disease
PPI	Proton pump inhibitor
miRNA	MicroRNA
DSS	Dextran sulfate sodium
IL-1β	Interleukin-1β
COX-2	Cyclooxygenase-2
STAT3	Transcription 3
ESCC	Esophageal squamous cell carcinoma
GAPDH	Glyceraldehyde-3-phosphate dehydrogenase
SE	Standard error
